# The *Trypanosoma cruzi* metacyclic-specific protein Met-III associates with the nucleolus and contains independent amino and carboxyl terminal targeting elements^☆^

**DOI:** 10.1016/j.ijpara.2006.11.016

**Published:** 2007-05

**Authors:** Eva Gluenz, Martin C. Taylor, John M. Kelly

**Affiliations:** Department of Infectious and Tropical Diseases, London School of Hygiene and Tropical Medicine, Keppel Street, London WC1E 7HT, UK

**Keywords:** *Trypanosoma cruzi*, Metacyclogenesis, Nucleolus, Gene knockout

## Abstract

Metacyclogenesis in *Trypanosoma cruzi* involves the differentiation of replicating non-infective epimastigotes into non-replicating metacyclic trypomastigotes. This pre-adapts parasites for infection of the mammalian host and is characterised by several morphological changes and structural alterations to the nucleus, including nucleolar disaggregation. Experimental investigation of these developmental processes has been hampered by a lack of robust molecular markers. Here, we describe the precise temporal expression of the *T. cruzi*-specific protein Met-III, in the genome reference strain CL Brener. Expression is restricted to metacyclics in the insect stages of the life-cycle and is rapidly down-regulated following invasion of mammalian cells. Met-III localises to dispersed foci typical of the disassembled nucleolus in metacyclics and to the discrete single nucleolus of cells soon after macrophage invasion. To identify elements that target Met-III, we generated a series of tagged green fluorescent protein fusion proteins and examined their sub-nuclear location in transformed parasites. These experiments demonstrated that amino and carboxyl terminal fragments, characterised by clusters of basic residues, could independently mediate nucleolar sequestration. To investigate the function of *Met-III*, we used gene deletion. This showed that Met-III is not required for the development of metacyclic trypomastigotes and that null mutants can complete the life-cycle in vitro.

## 1 Introduction

The insect-transmitted protozoan *Trypanosoma cruzi* is the causative agent of Chagas disease, the most important parasitic infection in South America (Tyler and Miles, 2003). During cyclical transmission by hematophagous triatomine bugs (Hemiptera: Reduviidae), the parasite goes through a series of life-cycle stages. Each stage is characterised by a distinct cellular morphology (Brener, 1973; Tyler and Engman, 2000; Elias et al., 2001) and development is concomitant with changes in metabolism (Adroher et al., 1988, 1990) and gene expression (Krieger et al., 1999). The basic features of the *T. cruzi* life-cycle have been known for nearly a century (Chagas, 1909). However, several crucial questions remain unanswered, including the number of distinct life-cycle stages, the nature of the signals that trigger differentiation and the point at which commitment occurs (Brack, 1968; Kollien and Schaub, 2000; Tyler and Engman, 2001).

Metacyclogenesis, the transformation of epimastigotes to metacyclic trypomastigotes, which occurs in the hindgut of the insect vector, is central to the *T. cruzi* life-cycle. It is required for the generation of parasites infective to the mammalian host. Several major phenotypic changes occur during metacyclogenesis, including non-proliferation, the development of infectivity, enhanced resistance to human serum and changes to nuclear organisation and cell morphology (Brack, 1968; Krieger et al., 1999; Tyler and Engman, 2000; Elias et al., 2001). During differentiation, the nucleus elongates and undergoes substantial ultrastructural changes. In TEMs of metacyclic nuclei, the nucleolus appears fragmented and the dense chromatin is dispersed, rather than being restricted to the nuclear periphery, as in epimastigotes (Elias et al., 2001). The molecular basis of these structural alterations is unknown, but nuclear reorganisation may be associated with the generalised transcriptional repression characteristic of the non-proliferative forms of the parasite. Attempts to dissect metacyclogenesis at the molecular level have been hampered by conflicting reports on conditions that might trigger or influence the process (Sullivan, 1982; Contreras et al., 1988), major differences between *T. cruzi* strains (Krassner et al., 1990) and a paucity of well-characterised stage-specific markers.

Recently, several genes have been identified that exhibit enhanced expression during metacyclic development in *T. cruzi* Dm28c (Avila et al., 2001; Dallagiovanna et al., 2001; Fragoso et al., 2003; Yamada-Ogatta et al., 2004). However, the biological functions, precise pattern of stage-specific expression and sub-cellular location of most of the corresponding gene products, remain to be defined. Furthermore, the possibility that they might have a direct role in parasite differentiation has not been addressed. Here, we have further investigated the properties of Met-III, a nuclear protein identified in a screen for transcripts that are up-regulated in metacyclics (Yamada-Ogatta et al., 2004). In the *T. cruzi* genome reference strain CL Brener, we show that Met-III is a specific marker for metacyclic trypomastigotes. It is rapidly down-regulated following invasion of mammalian cells and not expressed in bloodstream trypomastigotes. The Met-III protein is localised to the nucleolus and can be targeted to this sub-nuclear site by distinct amino and carboxyl terminal sequence elements.

## 2 Materials and methods

### 2.1 Cell culture

*Trypanosoma cruzi* CL Brener (Zingales et al., 1997) epimastigotes were grown at 28 °C in RPMI-1640 medium (Kendall et al., 1990). Metacyclic development was induced by addition of 20% Grace’s insect medium (Sullivan, 1982). Briefly, epimastigotes from a late logarithmic phase culture (0.8−1.2 × 10^7^ cells ml^−1^; <1% metacyclics) were collected by centrifugation and resuspended at the same density in 80% (v/v) fresh RPMI-1640 medium (as above) and 20% (v/v) Grace’s insect medium (Gibco BRL). To determine the percentage of metacyclic trypomastigotes, cells were stained with Giemsa and the morphology of >200 cells were scored by microscopic examination. In epimastigotes, the kinetoplast, the sub-organellar structure that contains the mitochondrial genome, is located anterior to the nucleus and has a tight disc-like configuration, whereas in metacyclics, it is positioned posterior to the nucleus and is more dispersed and spherical. Typically, 20% metacyclics were obtained 6–8 days after addition of Grace’s medium. Mouse macrophages (Raw 264) were used as hosts to generate mammalian stages of *T. cruzi*. Macrophages were grown in RPMI-1640 medium (as above, but without haemin) at 33 °C in a humidified 5% CO_2_ atmosphere in tissue culture flasks or 24-well microtiter plates. To obtain amastigotes for microscopic examination, macrophages were allowed to adhere to glass coverslips in 24-well plates in 0.5–1 ml medium for at least 2 h, before infection with 10 μl of a stationary phase *T. cruzi* culture. The culture supernatant was replaced with 1 ml of fresh medium every day until cover slips were processed for immunofluorescence. Cell-derived trypomastigotes were obtained by infecting 10 ml cultures of 50% confluent macrophage monolayers with a 100 μl parasite inoculum. Residual epimastigotes were removed by washing with medium after 24 h. Trypomastigotes first emerged from macrophages 6 days p.i.

### 2.2 Vector construction

To delete both *Met-III* alleles, flanking sequences were amplified from genomic DNA and fused to *Hyg* (gene encoding hygromycin phosphotransferase) and *Pac* (gene encoding puromycin acetyltransferase) selectable marker cassettes. The 5′ flank (416 bp) was amplified using primers _p_CCCgagctcTATGTGAGACTTGAACG_OH_ and _p_CCCgatatcTATTATTGTGTGACGCAGC_OH_. *Sac*I and *Eco*RV restrictions sites (lower case) were introduced to facilitate cloning. The 3′ flank (1.3 kb) was amplified using primers _p_CCCggtaccGAGTGCAATATATACAC_OH_ and _p_CCCctcgagTTTCTGCGCCAATTGAG_OH_ (*Kpn*I and *Xho*I sites in lower case). Vector pSHYGK was constructed by cloning *Hyg* between 450 bp of upstream and 750 bp of downstream sequence from *T. cruzi gGapdh* (Kelly et al., 1992). The *Met-III* 5′ flank was inserted into pSHYGK, upstream of this *Hyg* cassette. The 3′ flank was inserted downstream using *Xho*I and *Kpn*I. The *Hyg* gene in the resulting construct, pko-MET3-HYG, was replaced with *Pac* to obtain pko-MET3-PAC. For transfection, linear targeting cassettes were liberated from pko-MET3-HYG (4 kb) and pko-MET3-PAC (3.6 kb) with *Sac*I/*Kpn*I and gel separated from the plasmid backbone.

For expression of a green fluorescent protein (GFP)-tagged Met-III, the BPP-1-N fragment in pTEXeGFP-N (Bromley et al., 2004) was replaced with the *Met-IIIa* coding sequence. Primers _p_CCCgatatcATGTCGCGACGCAAAGATAAC_OH_ and _p_AAAggatccGGCCCACCTGTCCTCG_OH_ were used to amplify *Met-IIIa*, and the products digested with *Eco*RV and *Bam*HI. pTEXeGFP-N was linearised with *Eco*RI, blunt-ended and BPP-1-N released by *Bam*HI. The pTEXeGFP backbone and *Met-IIIa* fragment were ligated and the *Met-III-GFP* gene fusion verified by sequencing.

### 2.3 Transfection

Linear or circular DNA (∼10 μg) was added to 5 × 10^7^ log phase epimastigotes in 0.5 ml cytomix (2 mM ethylene glycol-bis(2-aminoethylether)-*N*,*N*,*N*′,*N*′-tetraacetic acid (EGTA), 120 mM KCl, 0.15 mM CaCl_2_, 10 mM K_2_HPO_4_/KH_2_PO_4_, 25 mM *N*-2-hydroxyethylpiperazine-*N*′-2-ethanesulfonic acid (Hepes), 5 mM MgCl_2_·6H_2_O, 0.5% glucose, 100 μg ml^−1^ BSA and 1 mM hypoxanthine; pH 7.6). Cells were electroporated using a Bio-Rad Gene Pulser with two pulses at 1.5 kV (25 μF). Transfected cells were selected over 6–8 weeks (G418 and hygromycin B, each at 80–100  μg ml^−1^; puromycin at 3–5 μg ml^−1^) and then cloned by limiting dilution. *T. cruzi* genomic DNA and RNA were prepared as described (Kelly, 1993). Transfected cell lines were analysed by Southern, northern and western blotting.

### 2.4 Generation of MET-III-specific antibodies

The *Met-IIIa* sequence was amplified from CL Brener genomic DNA using primers _p_AGAggatccGCAAGGATAACAAATCCCAC_OH_ and _p_CACaagcttGGCGCAGAAACTGCTA_OH_. The PCR product was inserted into the expression vector pTrcHis C (Invitrogen) using the *Bam*HI and *Hin*dIII sites, so that an amino terminal 6× histidine tag was fused in-frame with the coding sequence. Histidine-tagged Met-IIIa was expressed in *Escherichia coli* BL21 and purified on a nickel–nitrilotriacetic acid column (Qiagen) according to the manufacturer’s recommendations. To produce Met-III-specific antibodies, recombinant protein was inoculated into BALB/c mice as reported earlier (Wilkinson et al., 2002). Specificity of the antibodies was tested on Western blots of recombinant Met-III protein and *T. cruzi* cell lysates. Pre-immune serum from the same mouse served as negative controls.

### 2.5 Immunofluorescence microscopy

*Trypanosoma cruzi* epimastigotes, metacyclics and cell-derived trypomastigotes were fixed in suspension with 2% paraformaldehyde and dried on glass slides. Infected macrophages grown on glass coverslips were rinsed with PBS prior to fixation in 2% paraformaldehyde. Unreacted aldehyde groups were blocked with 50 mM NH_4_Cl and the parasites permeabilised with 0.1% saponin/PBS (PBS-S), with 50% horse serum. Cells were labelled with primary antibody for 1 h and secondary antibody for 30 min in PBS-S with 2% horse serum. After each incubation, slides were rinsed with PBS-S. Primary antibodies were anti-Met-III mouse polyclonal diluted 1:400 and L1C6 mouse monoclonal (a gift from K. Gull) diluted 1:200. Secondary antibodies were AlexaFluor 488- and 546-conjugated goat anti-mouse antibodies (Molecular Probes) diluted 1:400. DNA was stained with 1 μM TOTO-3 (Molecular Probes) in PBS/0.1% saponin/10 mg ml^−1^ RNase A for 20 min and slides were mounted in PBS/50% glycerol. Images were captured on a Zeiss LSM 510 axioplan confocal laser scanning microscope, processed with the Zeiss LSM Image Browser and assembled in Adobe Photoshop.

## 3 Results

### 3.1 Met-III as a specific marker for metacyclic T. cruzi

*Met-III* is a *T. cruzi*-specific gene, with two allelic variants (*Met-IIIa* and *Met-IIIb*) which share 97% identity in the genome reference clone CL Brener (www.genedb.org). This level of variation has been observed previously (Bromley et al., 2004) and is a consequence of the hybrid nature of this parasite clone (Machado and Ayala, 2001). At 573 nucleotides, both variants are shorter than the *T. cruzi* DM28c *Met-III* gene (Yamada-Ogatta et al., 2004), the result of a single nucleotide insertion at position 570. This moves the in-frame stop codon from position 598–600 (DM28c) to position 574–576.

**Fig. 1:**
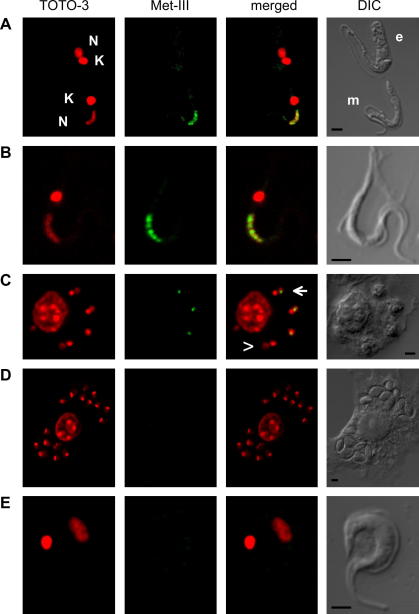
Localisation and stage-specific expression of Met-III. (A and B) Cells from a stationary phase culture were immuno-labelled with mouse antiserum raised against Met-III. This labels the nucleus of metacyclics (m) but not epimastigotes (e). The elongated metacyclic nucleus displays a dispersed Met-III signal (green), often concentrated in distinct foci. Kinetoplast (K) and nuclear DNA (N) are stained with TOTO-3 (red). (C–E) Met-III is down-regulated in the mammalian stages of the parasite. Mouse macrophages were infected in vitro with *Trypanosoma cruzi*. (C) Intracellular amastigotes, 24 h p.i. Three out of five intracellular parasites are still Met-III positive and the signal is restricted to a single subnuclear structure (arrow). Two intracellular parasites are Met-III-negative (arrowhead). (D) Intracellular amastigotes, 6 days p.i. (E) A trypomastigote released from a ruptured macrophage. Met-III-specific antibodies detected no protein in these cells. Scale bars = 2 μm.

Met-III expression has been shown to be up-regulated in metacyclics compared with epimastigotes (Yamada-Ogatta et al., 2004), but its expression in mammalian stages of the life-cycle has not been investigated. To determine if Met-III is exclusively expressed in metacyclics, antibodies raised against the Met-III protein (Section 2.4), were used to investigate all major life-cycle stages (epimastigotes, metacyclics, amastigotes and bloodstream trypomastigotes). This analysis showed that protein expression was restricted to metacyclics in the insect stages, where it localised to the nucleus (Fig. 1A and B). Met-III was absent from cells with an epimastigote morphology (defined by the relative positions of the nucleus and kinetoplast, Fig. 1A). Furthermore, we could also show that it is not detectable in intracellular amastigotes > 24 h p.i. of macrophages (Fig. 1D) or in cell-derived trypomastigotes (Fig. 1E). Occasionally, at early time-points p.i. (3–24 h), we observed Met-III labelling in some intracellular parasites (Fig. 1C). We conclude therefore, that Met-III is a tightly regulated metacyclic protein and that expression is rapidly curtailed following invasion of host cells.

### 3.2 Sub-nuclear localisation of Met-III

**Fig. 2:**
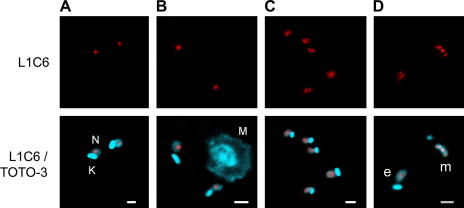
Dispersal of the nucleolus in non-replicating *Trypanosoma cruzi*. Fixed cells were labelled with the nucleolar-specific monoclonal antibody L1C6. In replicating cells, L1C6 staining (red) is confined to a small area of the nucleus: (A) exponentially growing epimastigotes; (B) amastigotes inside an infected macrophage. In non-replicating cells from stationary cultures, there are multiple dispersed foci of L1C6 staining: (C) stationary phase epimastigotes; (D) epimastigote (e) and metacyclic trypomastigote (m). Kinetoplast (K), nuclear DNA (N) and macrophage nuclear DNA (M) are stained with TOTO-3 (blue). Scale bars = 2 μm.

There was a striking sub-nuclear distribution of Met-III in many metacyclic cells, with the protein detected in distinct foci along the axis of the elongated nucleus (Fig. 1A and B). Other cells displayed a less punctuate, but never uniform, sub-nuclear distribution. Areas in the nucleus that labelled with the Met-III antiserum often stained weakly with the DNA-specific dye TOTO-3, tentatively suggesting a nucleolar location. Dispersal of the nucleolus in metacyclics has been reported (Elias et al., 2001). To assess this further, we used mAb L1C6, a nucleolar marker in *Trypanosoma brucei* (Durand-Dubief and Bastin, 2003; Li and Wang, 2006). In replicating *T. cruzi* (epimastigotes from exponentially growing cultures and intracellular amastigotes), this antibody identified the nucleolus as a single sub-nuclear structure coincident with weak TOTO-3 staining (Fig. 2A and B). Metacyclic cells, however, displayed a dispersed pattern of staining (Fig. 2D), similar to that observed with the Met-III-specific mouse antiserum (Fig. 1A and B). We also noted that dispersal of the nucleolar antigen occurred in all cells of a stationary phase culture (Fig. 2C), not just metacyclics. This dispersal therefore precedes other phenotypic events associated with metacyclogenesis, including repositioning of the kinetoplast and elongation of the nucleus.

**Fig. 3:**
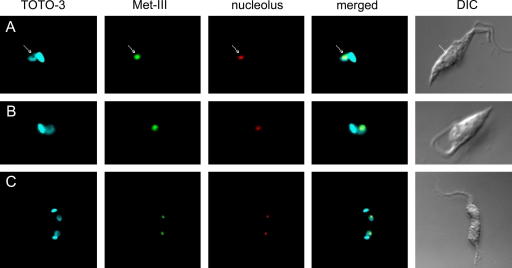
A Met-III-green fluorescent protein (GFP) fusion protein is targeted to the nucleolus in epimastigotes. *Trypanosoma cruzi* epimastigotes constitutively expressing a Met-III-GFP fusion protein from an episomal expression vector (Section 2.2) were fixed and analysed by fluorescence microscopy. Subcellular location of Met-III-GFP was determined by visualising GFP fluorescence (green). The nucleolus was labelled (red) with monoclonal antibody L1C6. Kinetoplast (K) and nuclear DNA (N) were stained with TOTO-3 (blue). Panel C shows a post-mitotic cell with two nuclei and two kinetoplasts.

To further investigate localisation, we constructed c-myc epitope- or GFP-tagged versions of Met-III. When these proteins were expressed in epimastigotes, they were targeted to the nucleolus. This was confirmed by co-localisation of the GFP fusion protein with the L1C6 nucleolar marker (Fig. 3). Expression of this normally metacyclic-specific protein in epimastigotes had no apparent effect on either the integrity of the nucleolus or the growth rate, and did not enhance the rate of development into metacyclics.

### 3.3 Nucleolar targeting of Met-III can be independently mediated by two distinct sequence elements

**Fig. 4:**
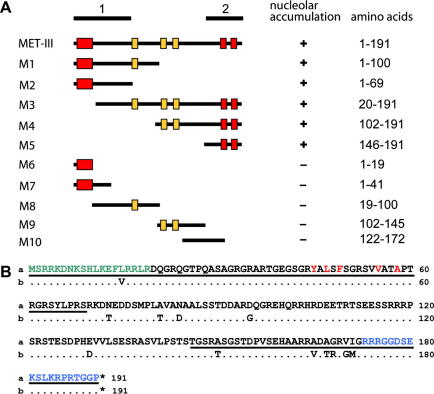
Constructs used to delineate Met-III nucleolar targeting signals. (A) Schematic representation of *Met-IIIa* fragments (M1-10) cloned upstream of *green fluorescent protein (GFP)* in a *Trypanosoma cruzi* expression vector (Section 2.2). The amino acids included in the fragments are indicated. Constructs were used to transfect epimastigotes and the resulting transformants examined by confocal microscopy (Fig. 5). Nucleolar targeting of the expressed fusion protein is indicated (+/−). Regions 1 and 2, found to confer targeting, are identified. Predicted nuclear localisation signals (NLSs) with a confirmed role in targeting are shown as red boxes (amino acid positions 3–19 and 173–187). Predicted NLSs that did not confer specific localisation are marked with yellow boxes (amino acids 67–72, 102–105 and 116–119). (B) Sequence of Met-III allelic variants from *T. cruzi* CL Brener. Differences between protein products are shown. Regions corresponding to elements 1 and 2 are underlined, with regions containing NLSs indicated in green and blue. Residues conforming to the consensus core sequence of the RNP-1 motif are shown in red.

**Fig. 5:**
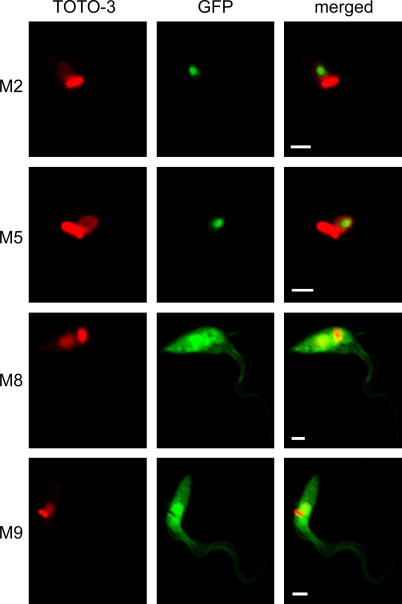
*Trypanosoma cruzi* epimastigotes expressing green fluorescent protein (GFP) fusion proteins tagged at the amino terminus with fragments of Met-III (Fig. 4) were fixed and examined by confocal microscopy. Fusion proteins can be visualised by green fluorescence, kinetoplast and nuclear DNA, by TOTO-3 staining (red). Fusion proteins M2 and M5 localised to the nucleolus, while M8 and M9 displayed non-specific distribution.

To investigate sequence elements in Met-III that facilitate sub-nuclear localisation, we generated 10 constructs (M1-10) in which fragments of the coding sequence were fused in-frame with a full-length GFP sequence (Fig. 4A and B). Each construct was expressed in stably transformed epimastigotes, where the nucleolus is a discrete entity and localisation is easier to interpret. The expressed fusion proteins fell into two distinct classes (Figs. 4 and 5). The first (M1-5) localised exclusively to the nucleolus in epimastigotes and displayed a more diffuse pattern in the elongated nucleus of metacyclics. This profile was identical to the full-length Met-III (Fig. 1). The second class (M5-10) exhibited a much more dispersed pattern, with the fusion proteins localised throughout the whole of the cell cytoplasm, including the flagellum and the entire nucleus, but excluding the kinetoplast. This pattern of distribution was the same as that for GFP only.

The results showed that at least two distinct elements from the amino and carboxyl termini of Met-III can independently mediate nucleolar targeting (Figs. 4 and 5). The 69 residue amino terminal fragment (construct M2) contains a predicted nuclear localisation signal (NLS) and a putative RNA binding domain (residues conforming to the consensus core sequence of the RNP-1 motif (Birney et al., 1993) shown in red, Fig. 4B). Exclusive nucleolar localisation was totally abolished when these two domains were separated (constructs M7 and M8). The smallest fragment to confer targeting was the 46 residue carboxyl terminal sequence (M5, Fig. 5). Twenty-seven of these residues were also present in the fragment expressed by construct M10, which did not mediate nucleolar localisation. The 19 residues specific to M5 contain two closely spaced NLS-like sequences. Similar results were obtained if Met-III was fused to a c-myc tag instead of GFP.

### 3.4 Generation and analysis of Met-III null mutants

**Fig. 6:**
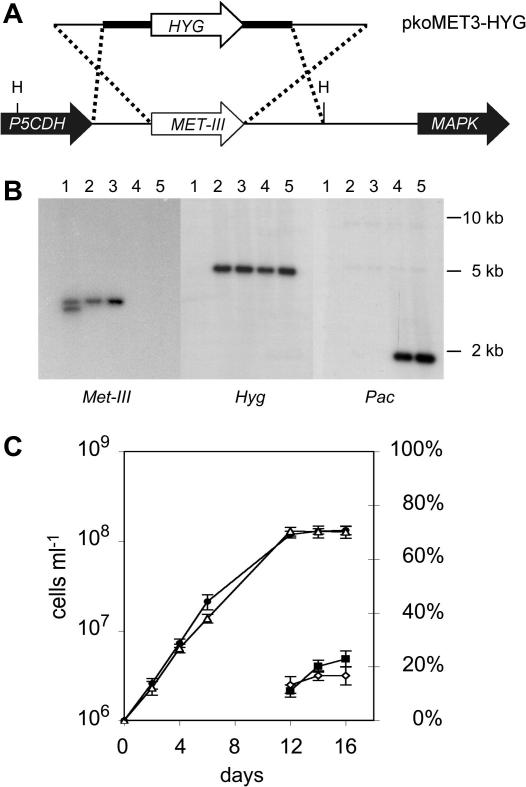
Generation and analysis of *Met-III* null mutants. (A) The *Met-III* genes were deleted by sequential integration of fragments containing the *Hyg* (as shown) and *Pac* selectable markers, with *Trypanosoma cruzi gGapdh* intergenic sequences (black) and *Met-III* flanking regions. The adjacent Δ-1-pyrroline-5-carboxylate dehydrogenase (*P5cdh*) and mitogen-activated protein kinase (*Mapk*) genes are indicated. H, *Hin*dIII restriction sites. (B) Southern blot of *Hin*dIII digested genomic DNA extracted from wild-type cells (lane 1), single-knockout cell lines generated by integration of the *Hyg* cassette (lanes 2 and 3), and double-knockout cell lines following integration with the analogous *Pac* construct (lanes 4 and 5). Hybridisation with *Met-III*, *Hyg* and *Pac* probes (as indicated) confirms the successive deletion of both *Met-III* alleles. (C) *Met-III* is not required for metacyclogenesis. Wild-type and *Met-III* null mutants were cultured for 6 days and then induced to differentiate in Grace’s medium for 10 days (Section 2.1). The cell density of wild-type (filled circle) and null mutants (open triangle) is plotted on the left axis. The percentage of differentiated cells, determined 6, 8 and 10 days after addition of Grace’s medium, is plotted on the secondary axis; wild-type (filled square); null mutants (open diamond). Each point shows the SD of the mean derived using eight measurements.

To test whether *Met-III* expression was essential for metacyclogenesis, null mutants were generated by successively replacing both alleles with drug-selectable marker genes (Fig. 6A and B). When null mutants were induced to differentiate in Grace’s insect medium, they entered stationary phase at the same time as wild-type cells (Fig. 6C). The subsequent development of metacyclic trypomastigotes in these stationary cultures occurred at a similar rate to wild-type (Fig. 6C). Morphologically, the mutants appeared normal in cells double-labelled with TOTO-3 and L1C6, and the timing and pattern of nucleolar dispersal was unchanged from that in stationary phase wild-type cells (not shown). To test if metacyclics derived from the double-knockouts retained infectivity, they were used to infect mouse macrophages in vitro. The mutants invaded macrophages, differentiated to amastigotes and then underwent replication. Trypomastigotes first emerged from lysed macrophages 6 days p.i., the same as wild-type cells. These results therefore show that expression of *Met-III* is not required for the development of metacyclics or for the completion of the parasite life-cycle in vitro.

## 4 Discussion

Little is known about the molecular mechanisms involved in *T. cruzi* metacyclogenesis. Research in this area has been hampered by the lack of well-characterised stage-specific markers, few of which are available for study of the *T. cruzi* life-cycle. Met-III has previously been shown to be up-regulated during differentiation from epimastigotes to metacyclics. Here, we tested all four major life-cycle stages and show that Met-III is highly expressed in metacyclics, and rapidly down-regulated following infection of mammalian cells. It is therefore a highly stage-specific marker and a useful tool for further molecular studies of the *T. cruzi* life-cycle.

Met-III localises to distinct foci in the metacyclic nucleus. Yamada-Ogatta et al. (2004) speculated that Met-III may be a component of dense chromatin. Based on the present study, we conclude that Met-III is associated with the nucleolus. The Met-III foci in the metacyclic nucleus (Fig. 1A and B) are typical of the dispersed pattern of nucleolar antigens, including L1C6 (Fig. 2D) and at early time points following invasion of mammalian cells the protein is associated with the discrete nucleolus (Fig. 1C). Moreover, when we expressed tagged versions of Met-III in epimastigotes, these proteins localised exclusively to the nucleolus (Fig. 3).

We also demonstrate that targeting of Met-III to the nucleolus can be mediated independently by two distinct sequence elements. There have been no previous reports on the signals that mediate nucleolar sequestration in *T. cruzi*. Generally, highly conserved sequences that function in nucleolar localisation have not been identified. There appears to be a diversity of signals across species that perform this role (Melese and Xue, 1995), although there are several instances where clusters of basic amino acids have been shown to be crucial (Hoek et al., 2000). Expression of GFP-fusion proteins in *T. cruzi* allowed us to identify amino and carboxyl regions that targeted Met-III to the nucleolus and conformed to this pattern. The amino terminal sequence contains a consensus bipartite NLS, which is two clusters of basic residues separated by a short (∼10) amino acid spacer (Fig. 4). In this case, however, nucleolar localisation was totally abolished when the NLS element was separated from an adjacent domain containing a putative RNA-binding motif, suggesting that these regions might act in concert to facilitate sequestration. Instances where nucleolar localisation is dependent on overlapping NLSs and RNA binding domains have been reported previously (LaCasse and Lefebvre, 1995). In the carboxyl terminal targeting region, an eight residue spacer separates two clusters of basic amino acids. There is some similarity between this Met-III fragment (construct M5, which contains the carboxyl terminal 46 amino acids) and a 32 amino acid domain that was sufficient for nucleolar targeting of *T. brucei* ESAG8 (Hoek et al., 2000). In both cases, these domains contained two clusters of basic residues, as well as some hydrophobic and acidic residues. Another similarity between Met-III and ESAG8 is that nuclear and nucleolar targeting could not be uncoupled. All fragments show either exclusive nucleolar targeting or non-specific localisation.

The function of Met-III in the metacyclic nucleus is currently unknown. In this life-cycle stage, the protein has a sub-nuclear distribution that mirrors the dispersed nucleolus, as judged by the antigen L1C6 (Figs. 1 and 2) (see also Elias et al., 2001). The nature and organisation of the disassembled nucleolus in metacyclics is poorly understood and there is no information on how the various components are dispersed. Our results clearly show that Met-III is non-essential for metacyclic development. This can be inferred from the phenotype of null mutants, which were found to have retained the ability to differentiate (Fig. 6C) and complete the entire life-cycle in vitro. Furthermore, neither episome-mediated expression of *Met-III* in epimastigotes, nor deletion of the *Met-III* gene, had any discernible effect on nuclear structure or dispersal of the nucleolus.

The literature on metacyclogenesis in *T. cruzi* contains many conflicting reports on conditions that induce differentiation from epimastigotes. Despite suggestions that cyclic AMP could play a critical role (Gonzales-Perdomo et al., 1998; Fraidenraich et al., 1993) and the efforts of numerous researchers (including ourselves), it has not been possible to unequivocally identify a specific factor that is both necessary and sufficient to trigger metacyclogenesis. The emerging consensus is that stress conditions, including nutrient depletion, favour differentiation, but that sufficient energy sources must be available to sustain the process (Tyler and Engman, 2000). In this study, we observed that nucleolar disruption and dispersal in *T. cruzi* appears to be a feature of all cells in a stationary phase culture, rather than just those that have differentiated to metacyclics (Fig. 2). The structural reorganisation of the nucleus correlates with a significant decrease in RNA polymerase I- and II-mediated transcription and protein synthesis (Elias et al., 2001) and the trigger for nucleolar dispersal could be nutrient depletion.

Research on a variety of organisms, corroborated by analysis of the nucleolar proteome (Scherl et al., 2002), shows that the nucleolus serves important functions beyond its more familiar roles in rRNA maturation and ribosome biogenesis. The nucleolus has a major influence on cell proliferation and gene silencing through sequestration of regulatory factors (Shou et al., 1999; Carmo-Fonseca et al., 2000) and functions as a cellular ‘stress sensor’ (Rubbi and Milner, 2003; Olson, 2004; Mayer et al., 2005). Alterations in nucleolar structure have been identified as a response to stresses that include heat shock, u.v. exposure, adenosine 5′-triphosphate/guanosine 5′-triphosphate depletion and hypoxia. The proposed mechanism implicates stress-mediated nucleolar disruption in the release of regulatory factors into the nucleoplasm (Olson, 2004). Given that cellular stress is the common denominator of conditions that promote metacyclogenesis in *T. cruzi*, and the temporal order of nucleolar dispersal and morphological differentiation, it is tempting to speculate that the trypanosome nucleolus may also act as a cellular ‘stress sensor’ and that nucleolar disassembly provides a crucial signal that primes cells for the initiation of metacyclogenesis.
